# Anticipatory Governance in Biobanking: Security and Risk Management in Digital Health

**DOI:** 10.1007/s11948-021-00305-w

**Published:** 2021-04-21

**Authors:** Dagmar Rychnovská

**Affiliations:** Department of International Relations, University of Sussex, Brighton, UK

**Keywords:** Biobanking, Biosecurity, Risk, Anticipation, Big data, Datafication of health

## Abstract

Although big-data research has met with multiple controversies in diverse fields, political and security implications of big data in life sciences have received less attention. This paper explores how threats and risks are anticipated and acted on in biobanking, which builds research repositories for biomedical samples and data. Focusing on the biggest harmonisation cluster of biomedical research in Europe, BBMRI-ERIC, the paper analyses different logics of risk in the anticipatory discourse on biobanking. Based on document analysis, interviews with ELSI experts, and field research, three types of framing of risk are reconstructed: data security, privacy, and data misuse. The paper finds that these logics downplay the broader social and political context and reflects on the limits of the practices of anticipatory governance in biobanking. It argues that this regime of governance can make it difficult for biobanks to address possible future challenges, such as access to biomedical data by authorities, pressures for integrating biobank data with other type of personal data, or their use for profiling beyond medical purposes. To address potential controversies and societal implications related to the use of big data in health research and medicine, the paper suggests to expand the vocabulary and practices of anticipatory governance, in the biobanking community and beyond.

We try to enhance the protection of data, and we forget to protect the individuals.^1^

## Introduction

Biobanking is becoming increasingly popular around the world. The US programme “All of Us” aims to collect medical and health data from more than 1 million volunteers to “accelerate health research and medical breakthroughs, enabling individualised prevention, treatment, and care” (National Institutes of Health, 2020). Chinese commercial biobank Zhangjiang Biobank seeks to gather 10 million samples, while the publicly-funded China Kadoorie Biobank has already collected over 500.000 samples, similar to public biobanks in the UK and Finland (Orchard-Webb, 2018). The largest biobank is located in Graz, Austria, storing over 20 million biomedical samples (Medizinische Universität Graz, 2020). Biobanking is particularly strong in Europe, where there are not only numerous large biobanks, but also intense collaboration among them, especially under the umbrella of BBMRI-ERIC (Biobanking and BioMolecular Resources Research Infrastructure—European Research Infrastructure Consortium). BBMRI-ERIC is a major international player in the field of biobanking. Funded by the European Commission, it works towards building a research infrastructure for the harmonisation and globalisation of biomedical research in Europe (BBMRI-ERIC, 2016). Despite the lack of a universally accepted definition, biobankers typically claim that the aim of biobanks is to act as a research repository and thus to collect, store and process biological samples and associated medical data and information (BBMRI-ERIC, 2016; Shaw et al., 2014).

In general, biobanking is situated at the intersection of two broader trends: big-data research and the datafication of health. However, despite many benefits, these trends raise diverse concerns. Big-data analysis has been heavily politicised due to numerous cases that show how this research can disrupt privacy and be exploited by for discriminatory purposes, human rights abuses, surveillance, or political gain (e.g., Klimburg-Witjes & Wentland, 2021; Sætnan et al., 2018). Similarly, the datafication of medicine and health raises questions about the potential misuse of big data due to the highly sensitive nature of medical and genetic data (Hoeyer et al., 2019; Rothstein, 2015; Ruckenstein & Schüll, 2017). Although the security of big data is discussed in diverse fields, discussion of biobanking as a specific area of big-data research is minimal. Debates on risk assessment and disaster recovery focus on mitigating risks (e.g., fires and flooding) to individual biobanks (Henderson et al., 2013; Simeon-Dubach et al., 2013), but discussions about societal risks related to the rise of organized collections of big medical data are surprisingly rare (Müller et al., 2020; O’Doherty et al., 2016; Sankar & Parker, 2017; Sariyar & Schlünder, 2019). At the same time, we can observe raising interest of states in the use of big medical data in political governance—not only for digital epidemiology, but also for policing and surveillance (Wee, 2020).

What can “go wrong” with biobanking? This paper opens up a debate on the political and security implications of organised collections of health data and biological samples and thus contributes to the research on the societal implications of biobanking and health data collections (e.g. Goisauf & Durnová, 2019; O’Doherty et al., 2016). Specifically, it aims to explore how threats and risks are anticipated and acted on in the governance of biobanking in Europe. For that purpose, it studies the discourse on ELSI (ethical, legal, and social implications) in biobanking, reconstructs the logics of risk that are present in this discourse, and reflects on the practices of anticipatory governance in biobanking. The research draws on studies of anticipatory governance (Aykut et al., 2019; Guston, 2014) and political science research on security and risk governance (Aradau et al., 2014; Huysmans, 2011; van Munster, 2005).

The paper first discusses the conceptual lens through which anticipatory governance of risks in biobanking is approached. Second, it outlines the political contexts in which biobanking evolved and briefly summarizes the controversies already dealt with in the field. Third, drawing on data from ethnographic research, interviews, and document analysis, the paper studies ELSI discourse on biobanking to reconstruct three logics of risk anticipation there: *data security*, *privacy*, and *data misuse*. The findings point to the prevalence of technical and legal framing of risks and threats and reluctance to engage with social and political context of biobanking research. To explore what inhibits addressing broader societal implications and vulnerabilities of biobanking, the paper discusses the practices of anticipatory governance in biobanking and argues that they dampen more critical reflection of the use of big-data research in digital health and medicine.

## Anticipatory Governance and Practices of (In)security

The governance of science and technology has been marked by increasing attempts to predict the future path of technology in the society (Nowotny, 2016). This endeavour has gradually become professionalised as *anticipatory governance*, which is understood as “a broad-based capacity extended through society that can act on a variety of inputs to manage emerging knowledge-based technologies while such management is still possible” (Guston, 2014: 219). The practices of anticipation focus on the future as an object of scientific inquiry and political intervention (Anderson, 2010). Anticipatory expertise involves a broad scope of stakeholders, from those engaged in foresighting to production ensemble (Guston, 2014: 218). Practices of anticipatory governance have been established mostly in the framework of technology assessment, research ethics, and responsible research and innovation.

This research looks at anticipatory governance as constituting a repertoire of meaning-making practices through which specific entanglements of science, technology, and society are constructed as risks. For that purpose, I turn to social scientific research on security, specifically critical security. Critical security studies make visible the inherently political nature of security, whether exercised as exceptional politics (logic of security) or as mundane, bureaucratic routines of risk management (logic of risk) (van Munster, 2005). It unpacks the meaning of security and problematises the assumptions that security practises draw on, including who or what is to be protected, from what or whom, how, by what means; who is to be in charge of making such decisions; who shall provide knowledge and expertise to inform these decisions; and whose voices are silenced in these decisions (Aradau et al., 2014). Critical security scholars also suggest that practices of security tend to hinder open democratic deliberation on specific social problem, by moving decision-making on insecurities to the hands of designated professionals (Buzan et al., 1998; Huysmans, 2011).

To study how threats and risks are anticipated and acted on in the governance of biobanking, I focus on the following elements in the discourse: *logic of risk* (how the insecurity is conceptualised and what logic of risk is involved), *referent object* (who or what shall be protected from an actual danger or a potential risk), *risk factors* (who or what is seen to contribute to the production of risks or dangers), *technologies of governance* (what measures are used to predict, manage and address these issues), and *social effects* (what subjects the technologies of governance construct and how they structure social relations). Before presenting the results of the research on diverse logics and technologies of risk that are mobilised in the governance of biobanking, biobanking and its controversies are introduced in more detail.

## Biobanking, Biosecurity, and Big-Data Controversies

Biobanking draws on the long history of medical data collection (De Souza & Greenspan, 2013), but in the context of digitalisation, (international) data sharing, and increasing interest in artificial intelligence-driven research, biobanks gain a new character as data repositories. Medical and pharmaceutical research drawing on a combination of basic health data, omics data, and lifestyle data shall lead to more personalised medical treatment of individuals and a better prediction of diseases. Biobanking is thus presented of as a key step towards personalised medicine (Ntai et al., 2014; Prainsack, 2017) and as crucial to the bioeconomy (Hauskeller & Beltrame, 2016) and health data economy (Tupasela et al., 2020).

Biobanking has the novel ability to gather unprecedented quantities as well as new quality of health data (specimens coupled with health records and lifestyle information). This, however, brings new challenges in terms of this data use and potential misuse. Since most biobanks are publicly owned (BBMRI, 2013), their governance shall be subject to public scrutiny.

Indeed, when biobanks became popular in late 1990s, they were met with a considerable suspicion. The most famous controversy was the attempt of the biopharmaceutical company deCODE to build, under an exclusive state licence from the Icelandic government, the first population-wide genomic biobank that would include data from the entire Icelandic population. Doctors, scientists, activists, and patients vocally resisted the initiative, criticising the presumed consent model and limited options for withdrawing from the state-sponsored yet privately operated biobank database, fearing breaches of privacy and commercialisation and exploitation of health data (BBMRI, 2013: 9; Winickoff, 2015). After several years of opposition and involvement of international experts, the initial contract was abandoned and the biobank resorted to the informed-consent model (Høyer, 2008). The field of biobanking has learnt many lessons from the Icelandic controversy, developed a new discourse about the value of biobanking for society, structured its practices in a more inclusive and collaborative spirit, and adopted the informed-consent model as a fundamental principle in biobanking governance (Pálsson, 2008).

Two decades later, there remain numerous concerns, yet biobank professionals focus mostly on ELSI issues, thus lagging behind discussions on (in)security of big-data research in other fields. Interestingly, European biobankers focus on biosecurity only in the context of biobanks storing pathogens (Müller et al., 2020), working thus with the understanding of biosecurity by the World Health Organization (WHO), which speaks about it in the context of “the protection of microbiological assets from theft, loss or diversion, which could lead to the inappropriate use of these agents to cause public health harm” (WHO, 2004: vii). In contrast, scientific and security authorities in the US start to pay attention to the potential security risks of big-data in life science (AAAS, 2014) and suggest that “Big Data in the life sciences is taking the biosecurity discussion beyond pathogens and toxins” (Kozminski, 2015: 3897). Emphasised in this regard are mostly the risks of illegitimate access (by unauthorised individuals) to big-data and the misuse of those data. However, others also raise concerns regarding the secondary use of health data (O’Doherty et al., 2016), such as the availability of biobanking data to governmental agencies, their use for other than health research purposes, or their integration with other type of data about participants and others (Sankar & Parker, 2017; Sariyar & Schlünder, 2019). How are these concerns reflected among biobanking professionals in Europe, and how do they anticipate future insecurities that this technology may bring about?

## Methodology

To investigate the anticipatory governance of risks and threats in biobanking, I turn to the ELSI discourse on biobanking in the community of European biobankers. Specifically, I explore the case of BBMRI-ERIC—a legal entity which brings together biobanks storing human biological data. BBMRI-ERIC is one of 21 European Research Infrastructure Consortia (ERICs) funded by the European Commission (European Commission, 2020) and representing cutting-edge European research. BBMRI-ERIC currently includes 20 member states and one international organisation, yet individual biobanks are also members. Established in 2013, it connects researchers, biobankers, industry, and patients to boost the exchange of biomedical data and research. BBMRI-ERIC works towards “making new treatments possible” and, to that end, it “shall establish, operate and develop a pan-European distributed research infrastructure of Biobanks and Biomolecular Resources in order to facilitate the access to resources as well as facilities and support high-quality biomolecular and medical research” (BBMRI-ERIC, 2016: art. 2.1). BBMRI-ERIC operates primarily on a non-economic basis and allows the research community in its member states access to its resources and services (BBMRI-ERIC, 2016: art. 3).

The study focuses specifically on ELSI Common Service group within the BBMRI-ERIC, which provides consultancy on ELSI to BBMRI-ERIC members and conducts research on ELSI themes. It can be seen as a site of knowledge production about social implications of biobanking and as a platform for sharing best practices in biobanking management. The data on ELSI discourse in BBMRI-ERIC have been generated in document analysis (BBMRI-ERIC documents such as position papers and reports), ten semi-structured interviews with ELSI Common Service experts from BBMRI-ERIC,^2^ and participant observations at the European Biobank Weeks 2018 and 2019—an annual conference bringing together medical professionals, private companies, researchers, ethicists and other stakeholders specialising in biobanking. The research participants were asked following questions: how long have they been in the field of biobanking and what agenda have they dealt with; what are the main future challenges to which the field of biobanking is preparing; how do biobankers think about what can go wrong with biobanking; through what processes do biobankers anticipate these future insecurities; how are these issues talked about in the community and at their home institutions at ELSI meetings; what measures are taken to prevent these risks and threats from happening; and which challenges are in their view overlooked. Participants were also offered to discuss any issue which they find relevant yet omitted in the interview.

In the coding of the data, I focused on what issues were identified in the data sample as risks or security concerns relevant for biobanking (*risk logics*). Then, drawing on frame analysis (Benford & Snow, 2000), I explored diagnostic frames, so who or what is seen as contributing to the actualization of the risk (*risk factors*) and who or what is seen as affected (*referent object*), and prognostic frames, so how are these risks supposed to be addressed, through what means and policies (*technologies of governance*), and which actors are involved in this and how (*social effects*). Based on the analysis, I reconstructed three logics of risk and compared the specific aspects of these rationalities. The findings are presented in the following section. Participant quotations are presented to illustrate the three security themes. I point out that while two risk rationalities are consistently and strongly present in the data, the third one—data misuse—is less pronounced. I then critically reflect on this finding in the context of the practices of anticipatory governance in the biobanking community.

The research can be read as a first inquiry into the anticipatory governance of risks in biobanking, but more data would be needed to explore the security and risk management in biobanking in more depth: for instance, no interviews were conducted with professionals responsible for the IT security in biobanking. Also, the discussion on ELSI issues in the BBMRI-ERIC might be different from how biobanks in different member states think about security and risk management. Finally, given the ongoing debate on GDPR and biobanking (e.g., Slokenberga et al., 2020), as well as new challenges related to engaging biobanking in the fight against the COVID-19 pandemic (Henderson et al., 2020), the discourse on risk in biobanking will likely evolve in the next years.

## Three Logics of Risk in the Anticipatory Governance of Biobanking

This section presents results of research on anticipating risks in the governance of biobanking, focusing on the case of BBMRI-ERIC. Three rationalities of risk were identified—data security, privacy, and data misuse. These logics are not mutually exclusive—while each one points to different aspects of biobanking and is managed via different measures, they can work together and can be mutually enforcing.

### Data Security

The most prevalent notion of future challenge in biobanking relates to data security, understood as a problem of securing the data stored in biobanks against unauthorised access,^3^ and building other barriers of access to biobanks. For instance, many interviewees equate the issue of threats and risks in biobanking with technical IT security practices, as exemplified by this interviewee:

That we would somehow extremely deal with this issue, that is not the case, but we know from experience in other countries what happened to the data when they were processed by the health service provider and there were hacking attacks, which I think could happen in the framework of biobanking, research may be distorted in the worst case, so it is actually a question of cyber security.^4^

IT security is also an area with the most explicit politics of risk assessment in biobanking. The notions of risks in the context of data security are most explicit, as they draw inspiration from other fields, including health service, and the broader societal discourse on cybersecurity. For ELSI experts, these risks are understood mostly as hacking, manipulation of data, and blackmail. The risk assessment conducted by the IT Common Service in BBMRI-ERIC is more elaborate, and in the area of (data) security it focuses on securing the data flow against threats such as malicious modification of data or code, information disclosure, and denial of service, and on threats associated with access to data (Holub & Common Service IT, 2016: 40).

The countermeasures against these risks are physical barriers, technological barriers and access regulation. These measures are perceived as reliable and uncontroversial, and overall, ELSI experts express great satisfaction with this technical form of risk management, without the need to deal with the issue further. For example, when asked about whether biobankers think about the risks that biobanking may pose, one interviewee argued

I would think so, but I have never heard that specifically. It is more about how do we ensure we are doing this lawfully. (…) I think that technical and security measures are very good. They have got a very good knowledge about quality, technology, data security. So, it is not about misuse, it is about making sure that they have got the lawful basis for the appropriate use. I think that is the bigger concern.^5^

This response is an example of a broader discursive pattern in the biobanking community, suggesting that the responsibility for security of biobanks lies in the technical domain, and that if IT and technical measures are followed properly, security does not need to be dealt with further. A similar response explicitly downplays the need for any additional attention to security:

[Security] is an issue because it is something that we need to take care of and is critical, it is important, but is not a problem. It should not interfere with the progress of biobanking. We should take care of it as we did [for] years and years and years in the hospital.^6^

The biobanking community perceives the protection of human samples and data stored in and shared across biobanks as a highly relevant yet technical issue. This rationality of risk draws on the notion of unauthorised (unlawful) access to biobanks as the main threat to the security of data, and it consequently encompasses a wide range of measures to protect the whole system (its physical as well as electronic component). This logic empowers actors like IT professionals and lawyers to identify threats, manage them, and design countermeasures. As long as everyone complies with the rules and procedures, the community does not feel the need to deal with the issue beyond the sphere of designated professionals.

### Privacy

When asked about what issues deserve more attention in biobanking, one interviewee responded that “privacy is the main concern.”^7^ Such responses are well represented in the ELSI literature about biobanking, documenting an ongoing controversy in the biobanking community about the best design for the contractual relationship between data participants and biobanks regarding the future use of the biosamples and data (e.g., Clayton, 2005; Murphy et al., 2009; Rothstein et al., 2016). The three mutually intertwined aspects of this controversy—technical, ethical, and legal—reflect layers of attempted control over the future use of biobanking data.

In BBMRI-ERIC, privacy is primarily dealt with as a matter of data protection, aimed at the protection of individual participants. For instance, in a risk assessment analysis, BBMRI-ERIC points to the threats of linkability and identifiability of data, content unawareness (i.e., that participants are unaware of what information is being shared about them), and policy/consent non-compliance (Holub & Common Service IT, 2016: 41). To address these problems, technical measures are developed and implemented at the BBMRI-ERIC level and promoted as best practices to individual biobanks.

In the realm of ethics and law, privacy also plays an important role. Specifically, ELSI experts debate how to balance the traditionally understood rights of research subjects (who shall be informed of the exact purpose and procedure of the research in which they participate) with the specificities of biobanks as research repositories for future studies rather than for particular, clearly defined research projects (e.g. Bledsoe, 2017; Lunshof et al., 2008). Protecting privacy is thus entangled with the uncertainty about future trajectories of the biobanking research—as one interviewee expressed, “we do not know now how we can help the participants, [or] what further information might be useful in future research studies.”^8^ The debate on protecting privacy reveals differing perspectives on the value of individual privacy in contrast to biobanking as a greater societal good bringing benefits for public health, and it has evolved towards prioritising the latter (Starkbaum & Felt, 2019).

The idea that biobanks participate in creating international, open-access genomic databases and thereby contribute to the universal benefit of producing knowledge on human genome is not new (Knoppers & Chadwick, 2005), yet the shift towards framing biobanking as a public good was most pronounced in negotiating the re-use of health data as the European General Data Protection Regulation (GDPR) was being prepared. The GDPR was initially seen as conflicting with the very idea of biobanks as repositories of personal data that will have multiple and unforeseen uses in the future. During negotiation of the regulation, biobanks (represented by the BBMRI-ERIC) were among the most vocal proponents calling for exceptions for researchers. The exception was eventually incorporated: data used for research purposes have a special status codified in GDPR article 89(1). As Starkbaum and Felt (2019) show, in discussions between biobank professionals (represented by the BBMRI-ERIC) and the EU, negligence of biobank data was presented as a key threat—biomedical researchers argued that the main value of biobanking lies not in its benefits to individual patients but in its potential benefits for public health.

This communitarian approach directly relates to the debate on designing informed consent for data participants. There are currently two main approaches to constructing consent: the first prioritizes anonymisation of data but it significantly decreases the quality of the data (especially the prospects of linking different type of data about participants) and their potential for biomedical research. The second calls for broad consent, which legitimizes the reuse of data in biobanks without the need to consult data subjects on specific use of their data. This approach is based on prioritising solidarity, mutuality, and the public value of biobanks, and shall be complemented by high transparency of biobanks and their openness to patient engagement (e.g., Gainotti et al., 2016). An alternative of broad consent is dynamic consent, which allows participants to track online the use of their data and biosamples and to opt out of certain areas of research (Prictor et al., 2018).

The technologies of privacy governance primarily refer to the individual as the referent object and to protection against risk factors, such as the disclosure of sensitive information or findings about an individual, and the use of participants’ data for undesired (i.e., not consented) research purposes. Simultaneously, though, the value of individual privacy is weighed against the collective benefits of biobanking. The risk of potentially violating individual privacy is framed as running against the risk of making biobanking-driven research ineffective and failing to deliver its promised benefits. This results in disputes about appropriate technologies of risk governance, especially ethical and legal, which is demonstrated by the unease with which GDPR is interpreted and implemented. Addressing privacy concerns openly empowers individual data participants and in turn incentivises biobanks to better demonstrate the benefits and security standards of their work to the public.

### Data Misuse

The last risk rationality covers rather marginal theme of biobanking as a source of insecurity to society, caused by the use of biobank data for unintended (and potentially harmful) purposes. This logic works (rather implicitly) with the state or third-party actor as the source of threat. This framing is rather vague, but some interviewees acknowledged that for some kinds of broader questions about the use of biobank data, there is no room in the current ELSI debate to address this particular issue. As one interviewee eloquently suggested,

Security is a bit like the elephant in the room. We are kind of not really addressing the issue. Maybe because we don’t really know what the security issues might be in five, ten, twenty years from now. Also, maybe because we probably do not want to acknowledge that there are security issues, so it is a way of reassuring ourselves that everything is fine. (…) [M]y experience is that each time we bring these issues to the table, we are kind of not totally ignored, but not taken seriously. Because if you look at the current situation, there have not been any large catastrophes yet that we can refer to. (…) It is very hard to foresee what to expect, and that is one of the reasons why biobankers are not really addressing this yet – because we don’t really know what to grasp.^9^

When asked about the specific trajectories of the overlooked misuse scenarios, experts mentioned the use of biobank data for some kind of genetic profiling of people, with a special focus on the rising role of artificial intelligence in biomedical research. For instance, one interviewee pointed out the importance of unpacking algorithms using our data that can serve for discriminatory purposes—whether for pricing, insurance, or other—and mentioned genetic profiling as a specific risk:

We need more transparency [about] how our data is used (…) It might be noble to do research, but the data is out [there] and the algorithms are there, so others can use those things to create discriminatory profiles, and they do not even need to apply them via machines, but with their brains… I am really a little bit concerned that nobody is aware of the problem.^10^

Another participant reflected on the role of political context in considering the risks of unintended use of data, and expressed their reservations against transfer of data and samples to the United States, as it does not have the same data protection laws as the EU, and data might be shared there with insurance companies or industry.^11^

Concerns about the misuse of profiling to discriminatory purposes are directly relevant for biobanking, since profiling is at the heart of personalised medicine (Nuffield Council on Bioethics, 2010). Profiling in general can be defined as “the deduction of information based on some characteristics of an individual or a group of individuals, which are either known beforehand or also deduced.” (Sariyar & Schlünder, 2019: 2). It thus contains three elements: “it has to be an automated form of processing; it has to be carried out on personal data; and the objective of the profiling must be to evaluate personal aspects about a natural person” (European Commission, 2018: 6–7). While “[g]enetic profiling is used in healthcare and biomedical research for associating genetic characteristics with increased or decreased likelihood of developing and overcoming certain diseases” (Sariyar & Schlünder, 2019: 3), medical profiling can be understood as “new services offering direct-to-consumer body imaging as a health check and personal genetic profiling for individual susceptibility to disease” (Nuffield Council on Bioethics, 2010: xvii).

A fairly overlooked actor in the anticipatory discourse on biobanking is the state, particularly with regard to its potential to exploit the data in biobanks. Yet some experts point out that the state is entitled to act with the aim of using biobank data for profiling—and in fact has already done so in several instances (O’Doherty et al., 2016). Albeit illegal under current terms of informed consent, there may be legitimate reasons for this intervention. The problem, as pointed out by one interviewee, is how this practice becomes entangled with the broader socio-political climate:

[Y]ou see the trends in Europe, and not only Europe, but worldwide, what is happening now in terms of politics, we see that nationalist groups are getting stronger, racist groups are getting stronger, so there is already risk that our data will be used for these kinds of purposes, because we are part of the world, we are not isolated. So that is a concern. And there are already examples of biobanks data being accessed by police or by courts for different purposes. And I would say that as long as you are in a democratic state with an independent justice and a fair trial system, you might say that this is fine, [though] consent-wise it is not, but the problem is that democracy is not written in stone, the political landscape is changing, so there are clear threats that this data can be misused in many differ way - for political purposes, for discriminatory purposes, for many different purposes that we cannot control.^12^

In sum, this logic of risk points to the secondary use of biobank data for discriminatory purposes (especially medical or genetic profiling) or other harmful purposes by the state or third-party actors as the threat. The referent object is rather implicitly the whole society, or parts of it, who might be subject to discriminatory or otherwise harmful treatment. The concrete measures that can be taken against this type of insecurity, beyond suggestions to discuss these issues in the field, are not considered, making the risks of misuse the “elephant in the room”.

## Anticipatory Governance and Its Limits

Table 1 compares the three logics of risk. While the first two are present in all types of analysed data and addressed in the community, the third rationality is rather vague and comparably underrepresented (and thus also incomplete in the analysis, as the table shows). Simultaneously, the third logic is the only one that considers broader socio-political conditions and the possibility of data/technology misuse. How and why is the problematisation of potential risks of biobanking to the society downplayed in the anticipatory discourse? This section argues that this omission can be situated first in the practices of anticipatory expertise in BBMRI-ERIC, and second in the emphasis on reliability and trust through which the field responds to existential concerns about the sustainability of the current biobanking model.

**Table 1:** Three logics of risk in the anticipatory governance of biobanking

Risk logic	Data security	Privacy	Data misuse
Referent object	Data and biosamples	Individual	Society
Risk factors	Disrupting the data flow; unauthorised access to data and biosamples; damage of data or samples	Insufficient data management leading to disclosure of sensitive information or findings about an individual; use of data for undesired research purposes	Secondary use of biobank data for discriminatory or other harmful purposes (e.g., medical and genetic profiling) by the state or a third-party actor
Technologies of governance	IT security measures; physical barriers in data centres; legal rules for data access and sharing	Informed consent; legal measures (GDPR and beyond); IT measures based on encryption and distributed responsibility (e.g. blockchain); public engagement	–
Social effects	Increasing role of technical experts; bureaucratisation of the risk governance	Empowerment of donors; biobanks motivated to persuade the public and policy-makers about the benefits and trustworthiness of biobanking	–

The focus of current risk management in biobanking is on protecting the biobank infrastructure against unauthorised access and use, and on designing the contract between individual participant and biobanks. These technologies of risk governance are based on the principles of individual responsibility, safety and compliance. On the contrary, political and security implications of biobanking do not belong to the main themes discussed at biobanking conferences or by the ELSI Common Service team. Instead, the ELSI experts focus more on practices of responsible research and prefer to talk about “awareness” as a call for attentiveness of biobank professionals to potentially undesired scenarios involving the biobank samples and research. The limited room for critique in the existing biobanking culture is a problem described already by O’Doherty et al., who observe that the ELSI practices and current safeguards in health data collections “focus on informed consent and anonymization, which are aimed at the protection of the individual research subject. They are not intended to address broader societal implications of health data and sample collections” (O’Doherty et al., 2016: 2).

Understanding the reluctance of the ELSI community to address the societal implications of biobanking and related security concerns, however, shall go beyond pointing the limits of current legal and technical measures or the lack of experience with serious data breach/ misuse scenarios. As one interviewee explains,

these kinds of [security] issues are very rarely discussed. And if you try to bring it up, it is very difficult… I think it is kind of taboo. … Because there is a lack of evidence, because it would be really threatening to our business, because what we are doing is intrinsically working with health data and samples, so if all of these security issues really exist and happen, then we do really have a problem. So that is why I think we like to assure ourselves that this is fine, that we have all the security systems in place and that it is not that scary.^13^

This neglect of bigger questions about the societal implications of biobanking and risks related to unintended use of biobanking data, however, is not surprising when considering how biobankers think about the future of the field in general. In fact, the key contemporary concern in the BBMRI-ERIC community, pronounced at conferences and in interviews, is how to ensure the sustainability of biobanking.^14^ The sustainability concern is a narrative of an existential threat to the very imaginary of biobanking as a public good that revolutionizes public health and contributes to the rise of precision medicine (cf. Aarden, 2017). On the one hand, biobankers complain that biobanks and their data are underused, and that a lot of metadata are missing from samples. On the other hand, the lack of progress of biobanking research contributes to increasing pressure from funders for more direct results of biobanking and for finding new ways to remain financially sustainable.^15^ The very model of biobanking in Europe is thus seen as under threat. To save it, biobankers seek ways to increase the benefits of this research infrastructure and ensure its scientific and financial sustainability. As argued by Biobank Graz researchers, “[s]ustainability of biobanks has to be realised on more than the financial level, since without the trust and participation of the public, every biobank would be empty” (Sargsyan et al., 2015: 419).

Taken together, biobanks seek to increase their potential to act as providers of a public good, yet dependent existentially on a structure of relations based on reliability and trust in biobanking. However, the pressure to act as a trustworthy and responsible partner may incentivise avoiding any controversies related to this research. This arguably diminishes the prospects of deliberating on broader and tougher questions, including the collaboration with private actors, or the conditions under which it would be legitimate to use biobank data for other than health research, for instance by state authorities. This anticipatory culture thus arguably dampens the prospects of caring for security and societal implications of biobanking more pro-actively (cf. de La Bellacasa, 2011), beyond the emphasis on individual responsibility, safety, and compliance with technical and legal standards.

## Conclusion

Security has been so far an overlooked layer in the discussion on biobanking, both in academic literature and in the professional field. The reasons for this lie arguably, first, in the anticipatory culture of the field, which pays attention to technical, legal, ethical, and participatory issues in biobanking governance, and, second, in more pressing concerns about the sustainability of the current model of biobanking. However, recent developments, including healthcare data breaches and integration of health data with other type of big data (as popularized during the COVID-19 pandemic), have made visible new types of use for, but also risks related to digital health data. Therefore, discussion about ethical, legal, and participatory aspects of biobanking—and digital health in general—cannot shy away from tending to security challenges as well, however distant they may appear.

This paper sought to open up the debate on security and biobanking and scrutinized how threats and risks are anticipated and acted on in the governance of biobanking. By studying the anticipatory discourse on biobanking in Europe, three logics of risk were reconstructed, focusing on the protection of data and biosamples from unauthorised access (*data security*), protection of individual privacy from the disclosure of sensitive information and from undesired research (*privacy*), and potential societal vulnerability to harmful or discriminatory secondary use of biobank data (*data misuse*).

The existing anticipatory practices, the language used to think about risks and threats, and the broader anticipatory culture of the community, however, might be insufficient to grasp the complexities of future challenges, including security risks. For instance, it remains unclear how the field might face dilemmas regarding demands to use the biobank data for “exceptional” reasons (e.g., criminal investigations, civil lawsuits) and how it will face commercialisation and potential pressures to new uses of data (e.g., rationing of health care resources, risk profiling of individuals). Therefore, we need to expand the vocabulary and practices of anticipatory governance so that we are able to discuss more openly the potential controversies related to the use of big data in health research and medicine, both in the biobanking community and beyond.

## Footnotes

1 Expert interview, 10th October 2019 (1).

2 Most of the ELSI Common Service experts were approached with the interview request and ca. half of them responded. Given the small size of this community, no further details about the interviewees are revealed in order to protect their anonymity. The interviews were conducted in person by the author. All interviews were conducted at a conference, except for one which was conducted at the workplace of the participant. The participants were familiarized with the goals of the research project and gave written informed consent. The interviews lasted on average 29 min, were audio recorded and bilateral only, and were fully or partly transcribed. On request, transcripts were returned to participants for corrections. The findings were not discussed with the interviewees.

3 For instance, BBMRI-ERIC works with this meaning in different contexts (https://www.bbmri-eric.eu/bbmri-eric/elsi-glossary). The IT Common Service of BBMRI-ELSI also works with “security” as a synonym for “data security” (cf. Holub & Common Service IT, 2016: 9).

4 Expert interview, 9th October 2019 (1).

5 Expert interview, 9th October 2019 (3).

6 Expert interview, 9th October 2019 (4).

7 Expert interview, 9th October 2019 (2).

8 Expert interview, 9th October 2019 (5).

9 Expert interview, 10th October 2019 (3).

10 Expert interview, 10th October 2019 (1).

11 Expert interview, 9th October 2019 (5).

12 Expert interview, 10th October 2019 (3).

13 Expert interview, 10th October 2019 (3).

14 The issue of sustainability was mentioned by almost all interviewees and was discussed at the relevant conferences.

15 Expert interview, 9th October 2019 (3).
